# Neonatal infection with *Helicobacter pylori* affects stomach and colon microbiome composition and gene expression in mice

**DOI:** 10.1128/iai.00250-25

**Published:** 2025-09-22

**Authors:** Katrine B. Graversen, Bella Bjarnov-Nicolau, Sigri Kløve, Krístina Halajová, Sandra B. Andersen

**Affiliations:** 1 Center for Evolutionary Hologenomics, Globe Institute, University of Copenhagen 552766, Copenhagen, Denmark; University of California San Diego School of Medicine, La Jolla, California, USA

**Keywords:** microbiome, early-life infection, *Helicobacter pylori*

## Abstract

The stomach bacterium *Helicobacter pylori* is estimated to infect half of the world’s population, and the health implications are affected by the age at infection. Neonatal *H. pylori* infection of mice is a relevant model to investigate metabolic and immunological effects. We performed an explorative study at the dynamic 1st month of life to compare the composition of the gastrointestinal tract microbiome and stomach gene expression of mice neonatally infected with *H. pylori* with that of uninfected mice. We found that *H. pylori* was present only in the stomach, and that *H. pylori* loads increase with age from 1 week after infection and onward, especially after weaning. Stomach and colon microbiome composition was strikingly similar between sites at the same sampling time but changed significantly over 1 week, with increased diversity at both sites. Despite the fact that the relative abundance of *H. pylori* in the stomach was low and never exceeded 3%, the composition and alpha diversity of the gastrointestinal microbiome was significantly affected by infection. In a pathway enrichment analysis, we found that stomach gene expression related to the extracellular matrix, muscle contraction, and metabolism was affected by infection. Expression of these key processes was, in infected mice, shifted away from that of control mice toward that of all mice sampled the subsequent week, which we speculate represents accelerated development in infected mice.

## INTRODUCTION

The evolutionary history and health implications of infection with the bacterium *Helicobacter pylori* make it a focus of experimental studies. Specialized on life in the acidic human stomach, *H. pylori* is estimated to infect half of the world’s population, with the association dating back 100,000–300,000 years (1, 2). It was discovered as the causative agent of stomach ulcers and cancer, where the disease risk is affected by factors such as host-microbe genomics, evolutionary match, and diet (3–5). The immune response to infection affects host development and response to other health challenges (1). In correlative human studies, *H. pylori* infection is associated with a lower risk of asthma and allergies (2–4). Mechanistic mouse experiments show that early-life infection has systemic anti-inflammatory effects through direct interaction with dendritic cells, promoting tolerance that can protect against disease (5, 6). The gastrointestinal microbiota also affects immune system development, and perturbations to the composition, particularly early in life, can cause lasting health effects. While *H. pylori* infection has been shown to affect the composition of the gastrointestinal microbiome in humans and mice (7–11), the effects of infection early in life, in particular, are not well characterized.

The environment of the gastrointestinal tract is highly dynamic in the early life of mammals and provides signals essential for immune system development (12). Sterile at birth, the tract is colonized by microbes from the mother and the environment, and the initial milk diet contributes maternal immune components, nutrients, and microbes that can metabolize them (13–15). Diversity in the intestinal microbiome increases as variety is introduced in the diet and stabilizes in humans at an adult level around the age of 3 years (16) and in mice at 3 to 4 weeks (17, 18). This triggers a strong immune response that facilitates appropriate future inflammatory responses (19). Transmission of *H. pylori* is primarily within families (20, 21), assumed via a gastro-oral or gastro-fecal route, and takes place in the 1st years of life where stomach pH and immune tolerance are higher (22). *H. pylori* has a range of adaptations to avoid the gastric acid, allowing it to thrive in the stomach niche (23). In contrast, it cannot compete against other microbes in the lower intestine, and in infected adults, *H. pylori* DNA can only rarely be detected in feces (24–26). When mice are infected with *H. pylori* in the 1st week of life, bacterial densities are initially low, followed by a high stable load after weaning with low grades of inflammation (5, 27). This is in contrast to adult infection, where inflammation is more extensive and final loads are lower (5, 27). As we believe early-life *H. pylori* infection of mice is a relevant model to investigate metabolic and immunological effects resembling human infection, we performed an explorative study to compare the gastrointestinal tract of mice in the 1st month of neonatal infection with *H. pylori* with that of uninfected individuals. We show that bacterial loads increase with age as expected and found that *H. pylori* cannot be detected in the lower intestinal tract early in infection. Despite low bacterial loads in the 2 weeks following *H. pylori* infection, the composition of the gastrointestinal microbiome was affected. Stomach gene expression related to the extracellular matrix, muscle contraction, and metabolism was altered in a way we speculate represents accelerated development.

## MATERIALS AND METHODS

### *H. pylori* culture

*H. pylori* inoculum for mouse infection was prepared as previously described (28). In short, a frozen stock of *H. pylori* strain PMSS1 was grown on tryptic soy agar (TSA) Sheep Blood plates (Thermo Fisher Scientific) for 72 h in microaerobic conditions. Colonies were swabbed into Brucella broth (BD Biosciences) with 10% fetal bovine serum (FBS, Sigma-Aldrich) and 0.06 mg/mL vancomycin (Sigma-Aldrich), and grown overnight with 100 rpm shaking. Cultures were diluted a few hours prior to use, and motility of bacteria was checked in the microscope. The liquid culture was centrifuged at 2,000 rpm for 10 min, and the pellet was resuspended in fresh Brucella broth with 10% FBS. The culture was plated in serial dilutions on TSA Sheep Blood plates to estimate colony-forming units (CFU) after 72 h growth, which was 2.4 × 10^9^–1.5 × 10^11^ CFU/mL.

### Animal experiment outline

Specific pathogen-free (SPF) C57BL6/J from Janvier (France) were bred with two females and one male per cage. Neonatal C57BL6/J mice were orally gavaged at 6 and 7 days of age with 50 µL of *H. pylori* PMSS1 culture (30 mice, five litters in three cages), or growth media alone as control (12 mice, two litters in the same cage). Groups of six mice were sacrificed 2 days or 1, 2, 3, or 4 weeks after gavage. We had fewer mice in the control group, and these were divided between the week 1 and week 2 time points (Table S1; Fig. S1A). At sacrifice, stomach and gut were dissected. The empty stomach was divided into an upper and lower section, approximately constituting the forestomach and the glandular part of the stomach. The upper and lower sections were cut into halves longitudinally for RNA and DNA extraction, respectively. The half sections for RNA extraction were immediately cut into small pieces (Fig. S1B). All stomach samples and approximately 1 cm pieces of small intestine and colon with content were snap-frozen on dry ice and stored at −70°C.

### DNA extraction

Total DNA was extracted from gastrointestinal tissue using the DNeasy Blood & Tissue Kit (Qiagen, Germany). One half of the upper and lower stomach sections, and small intestine tissue of *H. pylori*-infected mice, and one half of the lower stomach section of control mice from week 1 and 2 after gavage was used (Fig. S1A). The tissue was weighed, cut into small pieces, and put into 2 mL Eppendorf tubes together with 180 µL animal tissue lysis (ATL) buffer and one sterile aluminum bead. The tissue was mechanically lysed using the Qiagen TissueLyser for 40 seconds at 15 Hz. Samples were incubated with proteinase K at 56°C for 2 h with rotation, and DNA was extracted according to the manufacturer’s protocol. DNA was extracted from colon content from mice week 1 and 2 after gavage, with the Zymo Quick-DNA Fecal/Soil Microbe 96 Magbead Kit (Zymo Research, USA) according to the manufacturer’s protocol. A blank extraction control was included for stomach and colon samples.

### *H. pylori* quantification

For samples of infected mice, copy numbers of *H. pylori* from lower and upper stomach sections and small intestine were estimated by qPCR targeting the single-copy *H. pylori* gene glmM (28), in samples from day 2 to 4 weeks after gavage. A few control mice were included as negative controls. We used mastermix from qPCR BioSygreen (PCRBioSystems), and absolute copy numbers were calculated using a standard curve based on amplicons of diluted plasmid containing the glmM gene sequence (28). Absolute copy numbers were standardized to tissue weight. We tested whether there was a change in density over time in weeks with a linear model with location (upper or lower stomach) as a fixed effect.        

### Microbiome analyses

The V3-4 region of the 16S rRNA gene was sequenced from lower stomach tissue and colon content, from mice sacrificed 1 and 2 weeks after gavage. Library preparation and paired-end Illumina NovaSeq 6000 sequencing was performed at Novogene UK. Microbiome analyses were performed in RStudio (ver. 2023.12.1.402, [29]). Reads from the extraction controls were considered as contamination and used to clean the data using the *in silico* decontamination tool SCRuB (30). Reads were processed with the DADA2 pipeline (31). The reads were trimmed with truncLen = c(220,220) and merged with an overlap of 10 bp. Chimeras were removed and taxonomy assigned using the Silva reference database v.138.1 (32). In phyloseq (33), we removed chloroplast, mitochondria, and eukaryote sequences and pruned ASVs that occurred less than 10 times. ASVs were agglomerated using the tip_glom function with *h* = 0.03. phyloseq was also used for calculating the alpha diversity measured as the Shannon diversity index. The differences between groups at the different time points were determined by two-way analysis of variance (ANOVA) and Tukey’s *post hoc* test. Beta diversity was measured by the weighted UniFrac distance matrix, and statistical differences were calculated with the permutational multivariate analysis of variance (PERMANOVA) test in vegan (34). The MicroViz package was used to make the compositional barplot (35). Differentially present bacterial genera were identified with Linear Discriminant Analysis Effect Size (LeFSe) analysis using microbiomeMarker (36).

### RNA extraction and sequencing

RNA was extracted from one half of lower stomach tissue sections from weeks 1 and 2 after gavage with Qiagen MiRNeasy Tissue/Cells Advanced Mini Kit according to the manufacturer’s protocol. With a sterile aluminum bead, tissue was disrupted and homogenized in the Qiagen TissueLyser for 4 min at 25 Hz, and an additional DNase treatment was included. Samples were sequenced in two rounds on Illumina NovaSeq 6000, at the Department of Genomic Medicine, Rigshospitalet, Copenhagen, Denmark with TruSeq Stranded Total RNA library preparation, and at Novogene UK targeting all mRNA.

### Gene expression analysis

The RNA-sequencing data were trimmed to remove poly-G tails (--trim_poly_g) and filtered to remove duplicate reads with maximum accuracy (dup_calc_accuracy = 6) by fastP (37). It was processed using the nf-core/RNA-seq pipeline v.3.14.0 (38) with the default settings, aligning the reads to the provided mouse reference genome GRCm39 release no. 109 using STAR (39) and quantifying mapped reads using Salmon (40).

Principal coordinate analysis (PCA) and differential gene expression analysis were performed on a gene length-scaled count matrix with DESeq2 (41) in RStudio (ver. 2023.12.1.402 [29]), calculated using Wald statistics with Bonferroni corrections for multiple testing. Gene counts were first rounded to integers, and the count matrix was filtered to genes with counts >0 that were present in >10 samples. Two designs were applied in the analysis. To extract differentially expressed (DE) genes between *H. pylori* infected and controls across both time points, the design included treatment, time, and sequencing run (design = ~ Treatment + Time + Run) with contrast = c(“Treatment,” “Hp,” “Ctrl”). To extract DE genes with *P*_adj_ < 0.05 at each of the individual time points, a combined four-level factor consisting of treatment and time was included in the design (design = ~ Treatment_Time + Run) with contrast = c(“Treatment_Time,” “Hp_W1,” “Ctrl_W1”) and contrast = c(“Treatment_Time,” “Hp_W2,” “Ctrl_W2”). Statistical differences between treatments, sampling time, and sequencing run on overall gene expression were calculated by PERMANOVA test in vegan::adonis2 (34) based on Bray-Curtis dissimilarity matrix between samples.

To identify enriched pathways, we applied STRING (version 12.0; https://string-db.org/; [42]) on differentially expressed genes with a more permissive cutoff of *P*_adj_ < 0.1 from the DESeq2 results, for week 1, week 2, and across both weeks. We applied STRING to total gene lists. As background, we used all the genes with detected expression. Within STRING, we chose to report Reactome pathways (43) with a signal >1. The signal represents a weighted harmonic mean between the observed/expected ratio and -log(FalseDiscoveryRate).

## RESULTS

### *H. pylori* colonization

**Fig 1:**
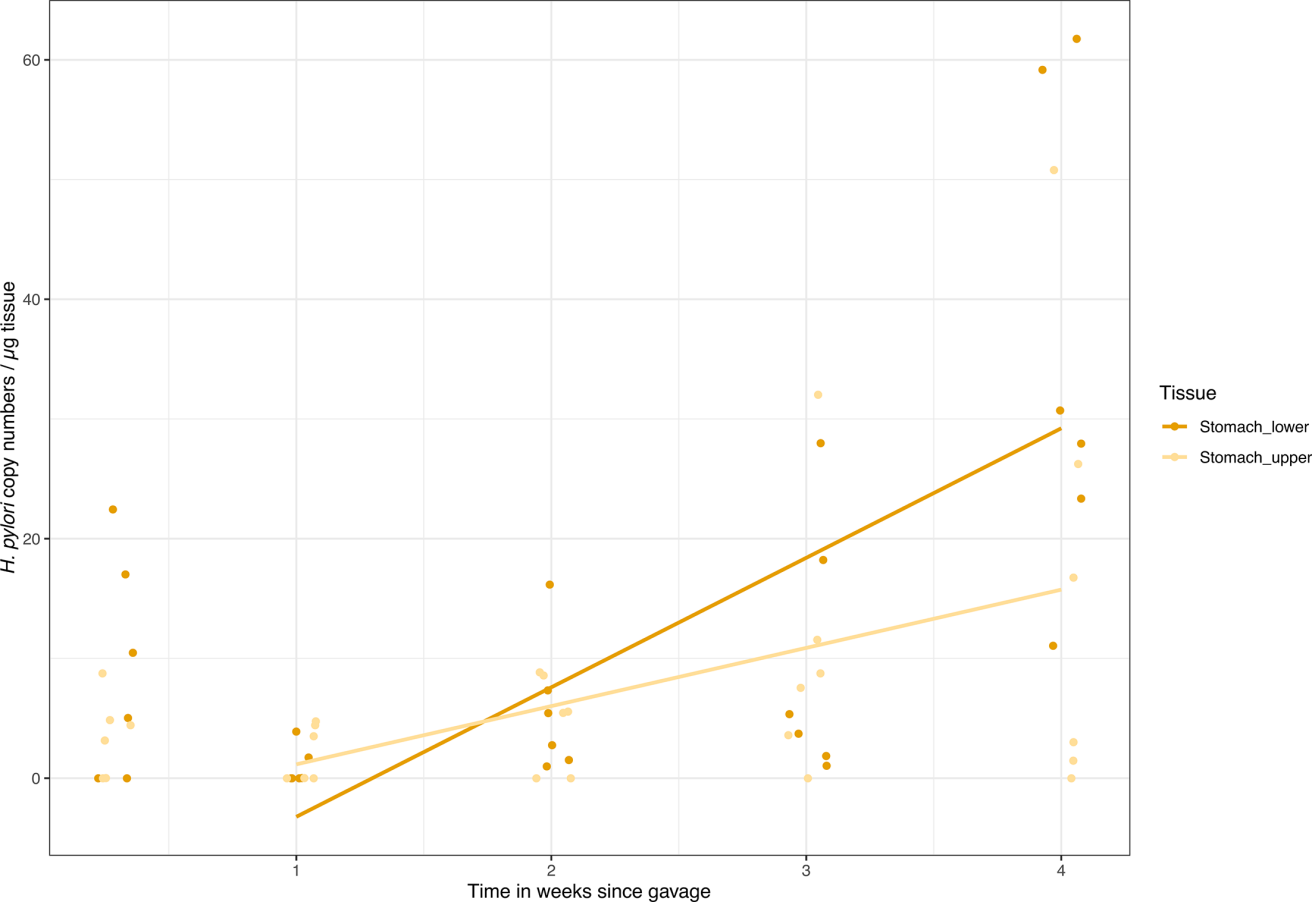
The *H. pylori* copy numbers per microgram tissue detected by qPCR increase significantly over time from week 1 to 4 weeks after gavage. From 2 days to 1 week after gavage, there was a drop in bacterial load, which likely represents wash-out of the inoculum. For this reason, the linear regression was performed from week 1. There was a trend toward a greater increase in bacterial load in the lower part of the stomach.

In stomachs, there was an initial drop in *H. pylori* loads from day 2 to 1 week post-gavage, likely representing the wash-out of the initial inoculum (Fig. 1). To account for this, linear regression on the change in bacterial load was performed on data from week 1 after gavage and onward. Here, we found a significant increase in *H. pylori* counts adjusted for tissue weight (Fig. 1; linear regression, *F* = 10.26, *df* = 3, 44; *R*^2^_adj_ = 0.37; *P*_adj_ < 0.001). There was a non-significant trend for higher loads in the lower stomach compared to upper stomach (tissue location: *t* = −1.91; *P* = 0.062). No *H. pylori* was detected in small intestine samples (data not shown).

### 16S data processing

For the colon samples, we obtained a median value of 64.8 k ± 2.3 k median absolute deviation (MAD) raw reads, with the corresponding values for the stomach samples being 66.2 k ± 4.8 k MAD raw reads. Following filtering, denoising, merging of paired reads, and chimera removal, this was reduced to 50.1 k ± 4.2 k MAD and 58.5 k ± 5.3 k MAD reads, respectively. Rarefaction curves confirmed that the sequencing depth was adequate (Fig. S2). The negative controls had a sequencing depth of 1.8 k and 7.3 reads. A total of 2.6 M sequences were classified as 2,924 ASVs, which were reduced to 575 ASVs following removal of chloroplast, mitochondria, and eukaryota ASVs, as well as pruning and agglomeration.

### Microbiome composition analyses

**Fig 2:**
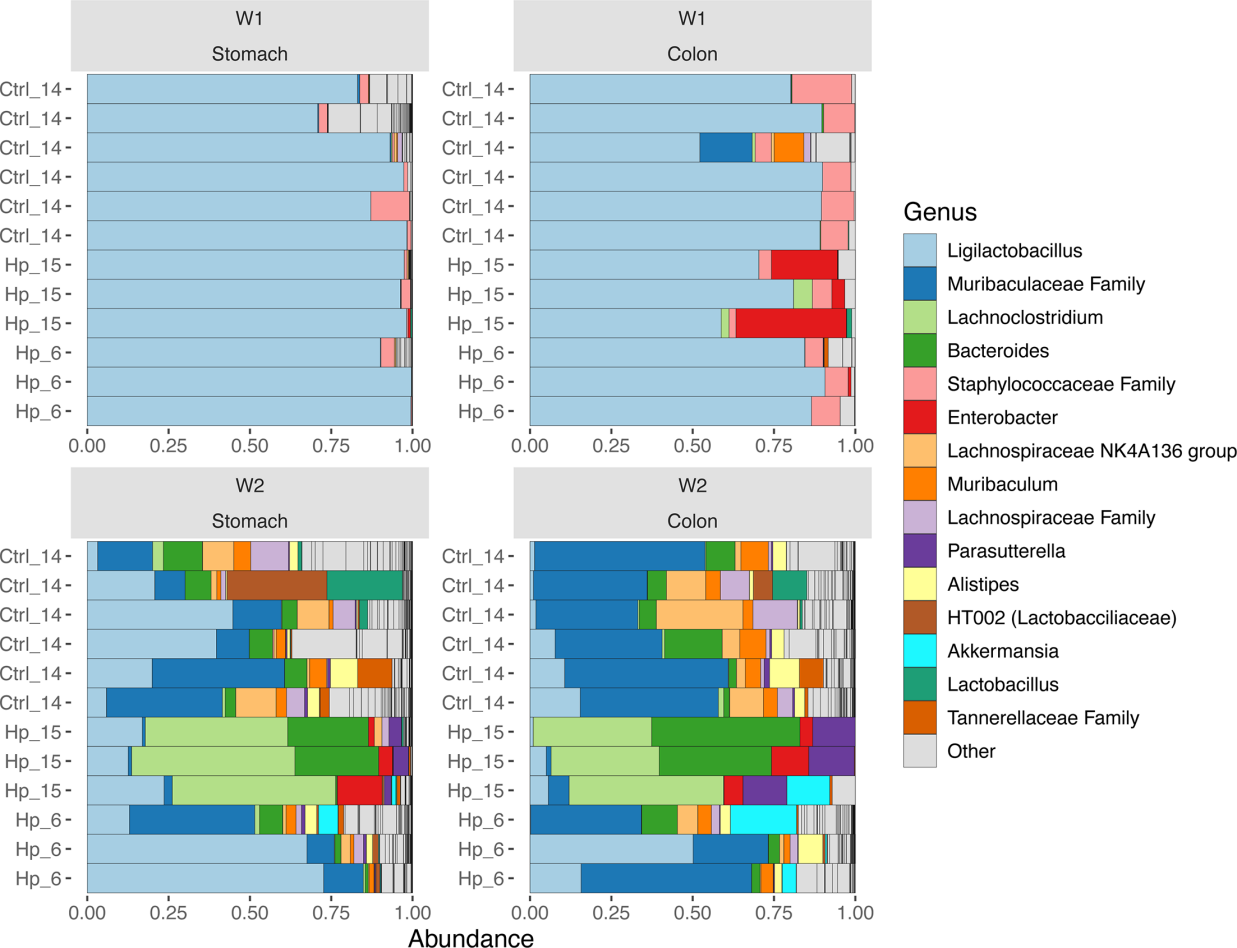
Distribution of the 15 most abundant genera in the stomach and colon samples, sampled 1 week (W1) and 2 weeks (W2) after gavage. Samples are denoted by treatment, which is either gavage with a control (Ctrl) solution or *H. pylori* (Hp) culture, and litter is indicated by number.

**Fig 3:**
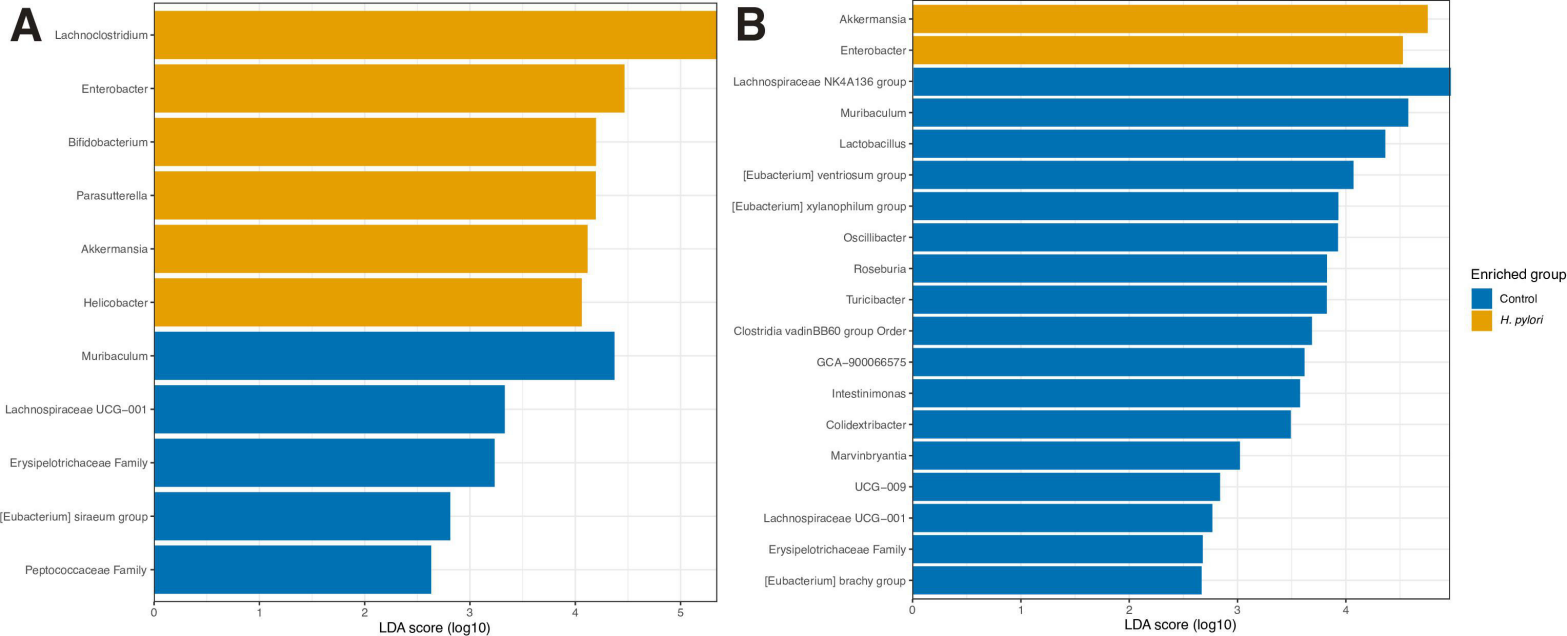
Genera found to be differentially present in control (blue) or *H. pylori* (yellow) samples. (**A**) Stomach and (**B**) Colon samples from 2 weeks after gavage by LEfSe. On the *x*-axis is the effect size score given by linear discriminant analysis.

**Fig 4:**
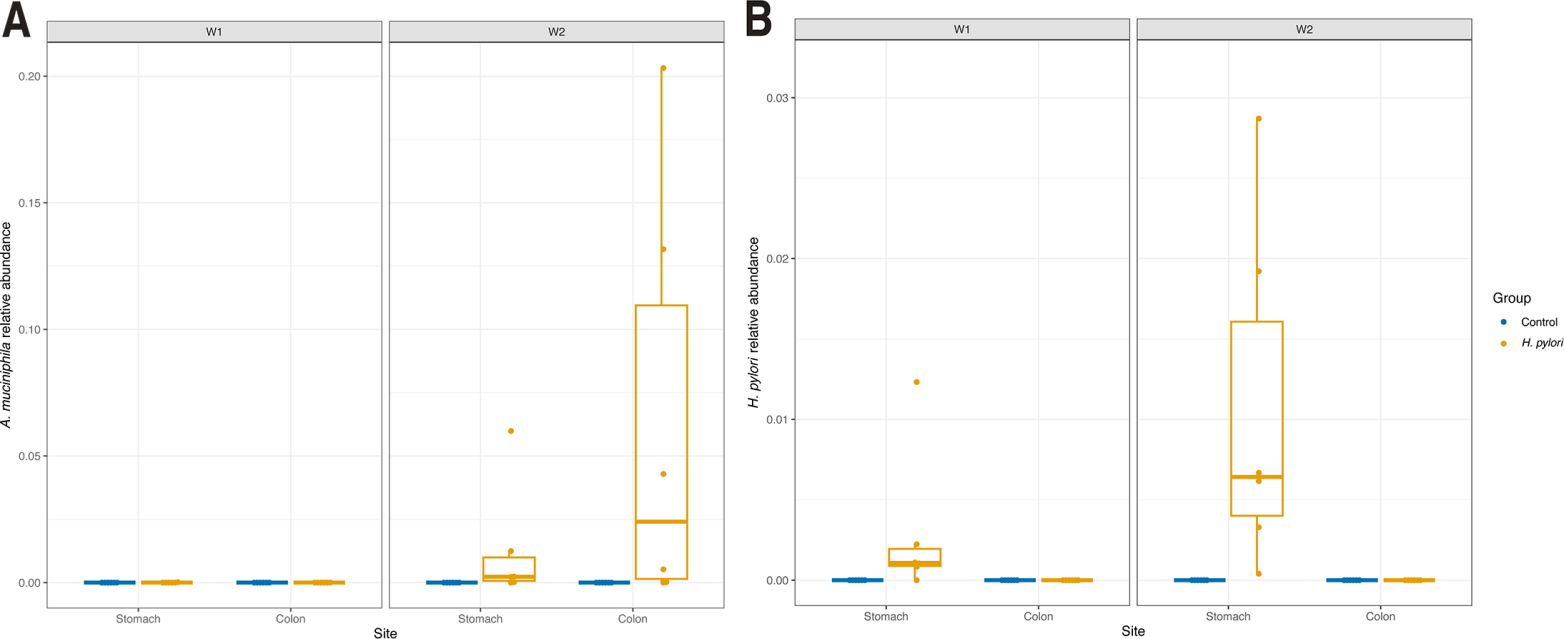
Relative abundance of (**A**) *Akkermansia muciniphila* and (**B**) *Helicobacter pylori* in stomach and colon samples from week 1 (W1) and week 2 (W2) after gavage, depicting mice infected with *H. pylori* in yellow and control animals in blue.

We visualized the microbiome composition in stomach and colons samples collected 1 and 2 weeks after gavage by depicting the relative abundances of the top 15 most abundant taxa at genus level. Strikingly, the stomach and colon samples from the same week were more similar to each other than to those from the same site collected the other week (Fig. 2). Samples from week 1 were dominated by *Ligilactobacillus* lactic acid bacteria, while a *Muribaculaceae* genus expanded in week 2. In week 1, the LEfSe analyses showed *H. pylori* to be enriched in the stomach of infected mice and *Enterobacter* in the colon, which was specific to litter 15. In the control mice, Lachnospiraceae family was enriched in the stomach and *Bacteroides* in the colon in week 1 (data not shown). In week 2, the *H. pylori*-infected mice from litter 15 with *Enterobacter* experienced an additional bloom of *Lachnoclostridium*, which came out as significantly enriched genera in the stomach in the LEfSe analysis (Fig. 3). *Akkermansia* was significantly more abundant in both stomach and colon samples in week 2 in *H. pylori*-infected mice (Fig. 4A). *Helicobacter* was present only in stomach samples of infected individuals, and even here only reached a median prevalence of 0.11% in week 1 and 0.64% in week 2 (Fig. 4B). Based on the compositional plots, we analyzed the alpha and beta diversity for each week separately.

### *H. pylori* infection lowers alpha diversity

**Fig 5:**
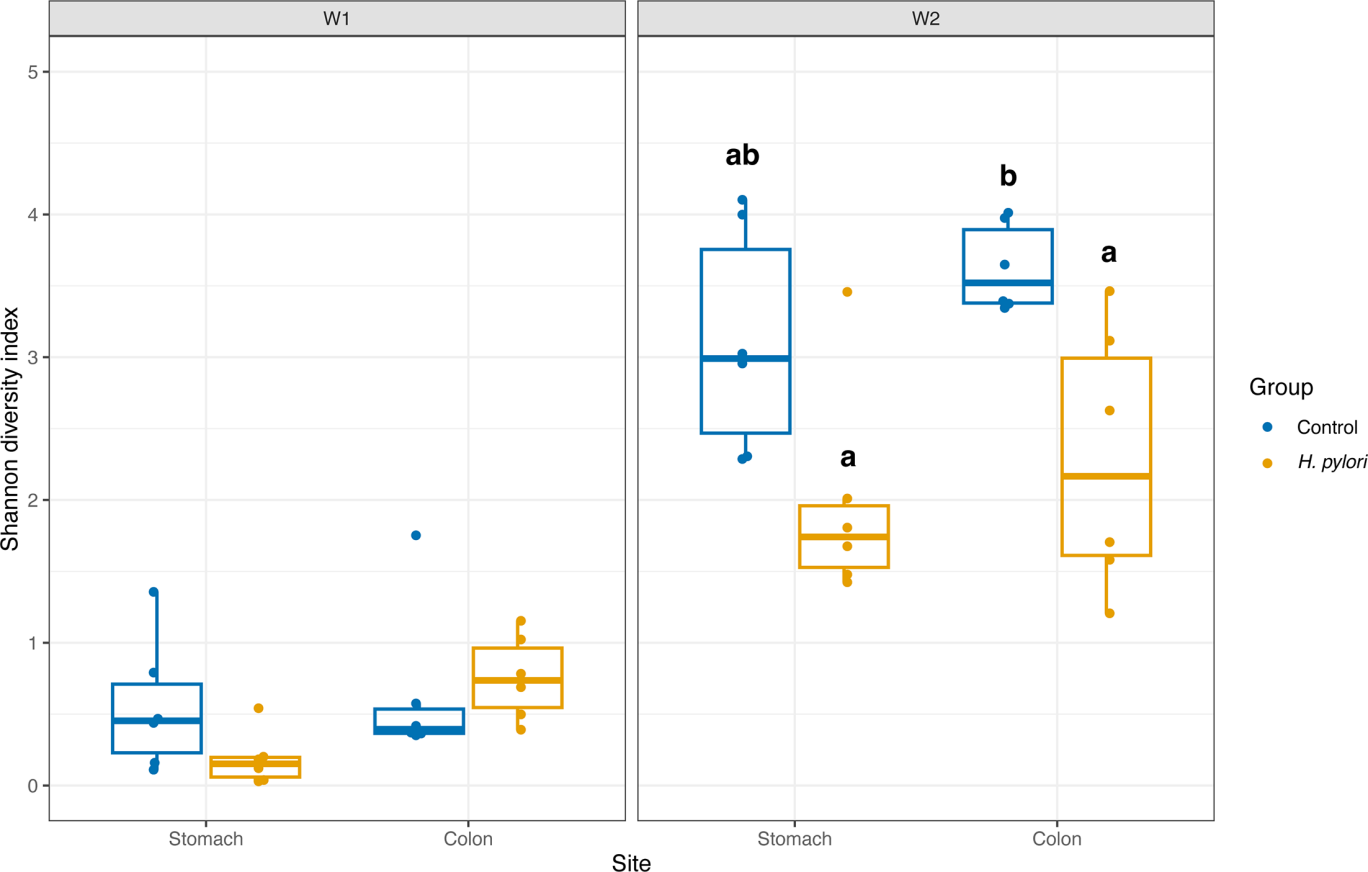
Alpha diversity measured by Shannon diversity index of gastrointestinal samples from early-life *H. pylori*-infected mice compared to controls. Samples were taken from stomachs and colons of mice that were sacrificed 1 (W1) and 2 weeks (W2) after gavage. Letters indicate groups that have significantly different means at the 0.05 level following two-way ANOVA and Tukey honestly significant difference (HSD). The boxes depict median values and interquartile range, and whiskers depict the minimum and maximum values, with individual data points as dots.

The alpha diversity, measured as the Shannon diversity index, increased from week 1 to week 2 (Fig. 5; Table 1). In week 1, there were no significant differences between treatments, but a trend for lower diversity in the stomachs compared to colons for the Shannon index (Table 1). In week 2, diversity was lower in samples from *H. pylori*-infected mice and significantly lower in the colon of infected mice compared to control colons (*P*_adj_ = 0.022), with the same trend for the stomach (*P*_adj_ = 0.061).

**TABLE 1:** Two-way ANOVA results comparing the alpha diversity, estimated by the Shannon diversity index, of colon and stomach samples of mice infected with *H. pylori* and controls^*a*^

	Week 1		Week 2	
	*P*-value	*F*-value	*P*-value	*F*-value
Group	0.452	0.587	0.000484	17.302
Site	0.059 .	4.013	0.184	1.892
Group:site	0.153	2.207	0.735	0.118

^*a*^ The analyses were performed for week 1 and week 2 alpha diversity independently, and in week 2, there was a significant difference in alpha diversity between *H. pylori* and control mice.

### Beta diversity plot shows differences in phylogenetic profiles

**Fig 6:**
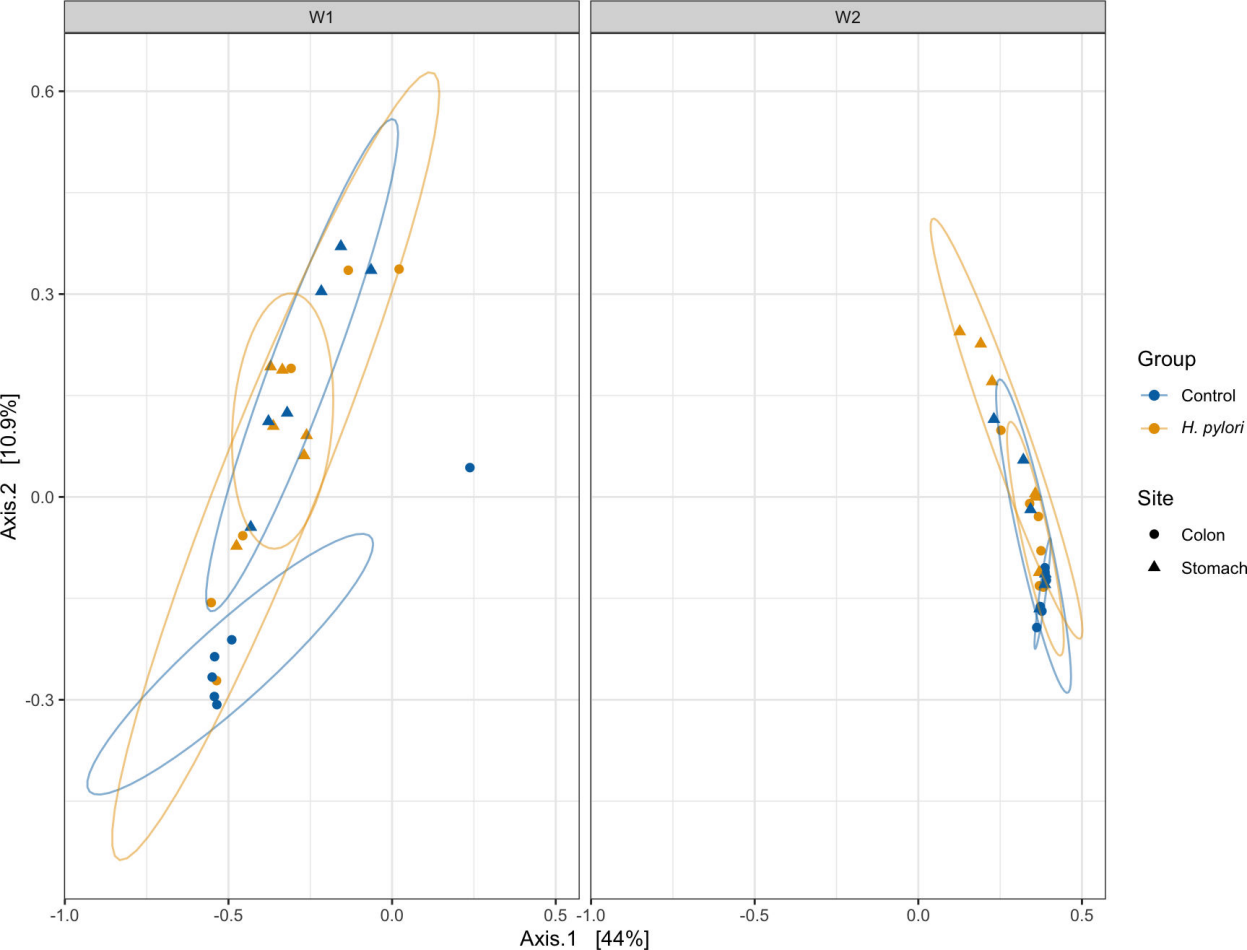
Weighted UniFrac distances depicting beta diversity of colon (circles) and stomach (triangles) samples from early-life *H. pylori* infection in mice, collected 1 week (W1) or 2 weeks (W2) after gavage with *H. pylori* (yellow) or a control solution (blue).

To further analyze the differences in the microbial composition of our samples, we looked at sample-to-sample similarities in a beta diversity plot of weighted UniFrac distances that relies on phylogenetic distances (Fig. 6). In week 1, there was a significant difference between sites. In week 2, there were significant differences between sites and treatments and a trend toward an interaction (Table 2).

**TABLE 2:** PERMANOVA results from analyses of beta diversity, estimated by Weighted UniFrac distance, of colon and stomach samples of mice infected with *H. pylori* and controls^*a*^

	Week 1			Week 2		
	*F*-value	*P*-value	*R* ^2^	F-value	*P*-value	*R* ^2^
Group	5.3173	0.001	0.193	4.4913	0.001	0.1583
Site	0.9048	0.544	0.0328	2.2958	0.013	0.08092
Group:site	1.3643	0.121	0.0495	1.5841	0.078	0.05583

^*a*^ In both weeks, there were significant differences between samples from *H. pylori*-infected mice and controls, explaining 15.8%–19.3% of the variation in the data, and in week 2, there was also a significant difference between samples from colon and stomachs, explaining 8% of the variation.

### Transcriptome data processing

The data set from Rigshospitalet had a total of 1,215.6 M reads, with a median of 98.9 ± 35.3 M MAD reads per sample. In the Novogene data set, there were a total of 457 M reads, with a median of 40.4 ± 11 M MAD reads per sample. After the initial fastP trimming, the data set from Rigshospitalet had a total of 854.2 M reads, with a median of 64.8 ± 6.2 M MAD reads per sample. In the Novogene data set, there were a total of 403.4 M reads, with a median of 30.9 ± 1.7 M MAD reads per sample. This was reduced to 341.2 M reads following nf-core processing with a median of 13.8 ± 2.4 M MAD reads per sample, where the largest fraction of reads was removed in the deduplication step.

### Gene expression analysis

**Fig 7:**
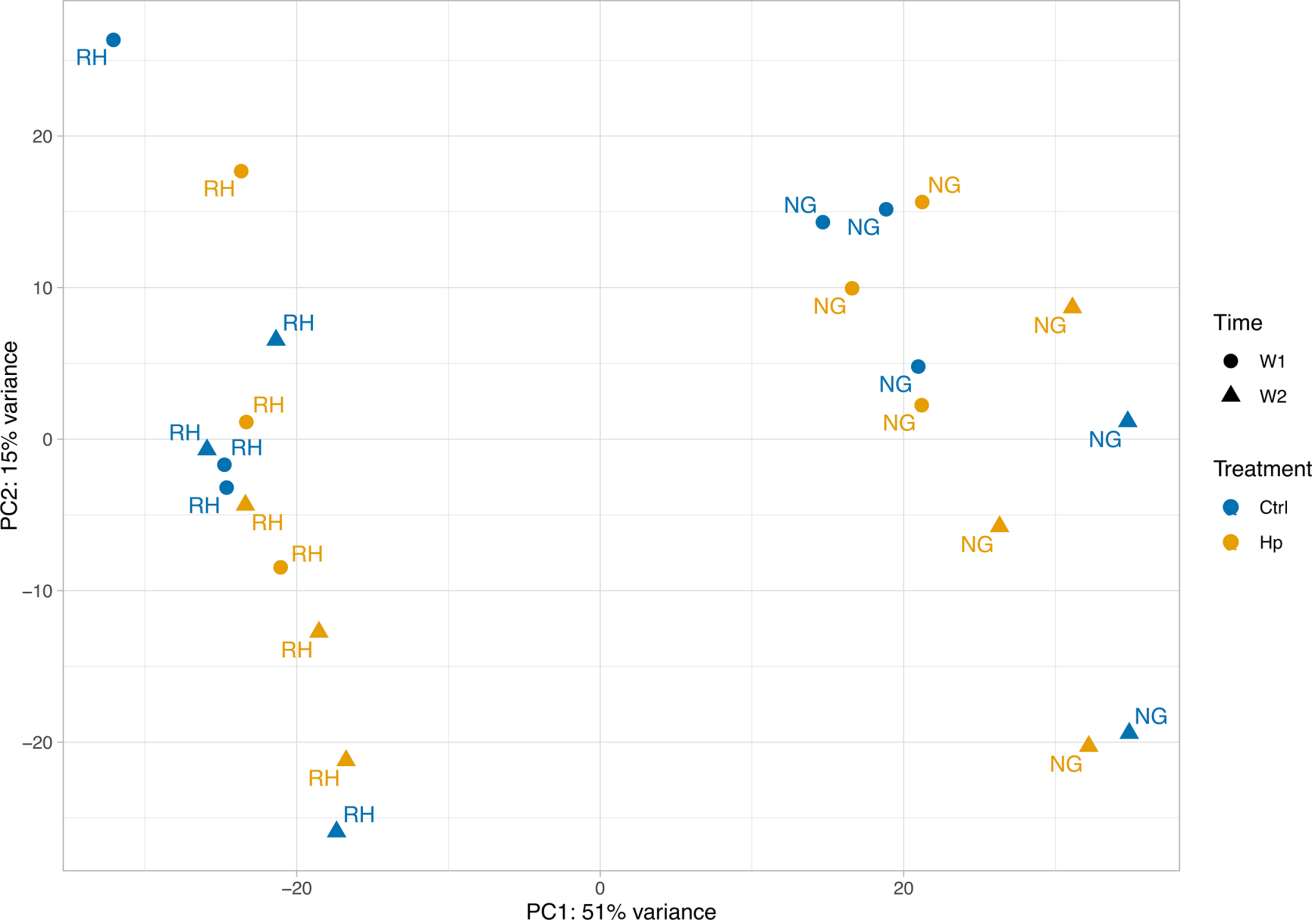
PCA plot of stomach RNA-sequencing data from early-life control or *H. pylori*-infected mice illustrating differences in sequencing run (RH, Rigshospitalet; NG, Novogene), time (circles: week 1 samples, triangles: week 2 samples), and treatment (blue: control, yellow: *H. pylori*).

A total of 22,379 genes were found to be expressed in our stomach tissue samples. A PCA of expression profiles from the DESeq2 results shows a clear separation between sequencing run (labels) along PC1, with 51% of the variance explained along this axis (Fig. 7). There was a significant difference between sampling times and sequencing run (Table 3). Despite no clear separation by treatment on the PCA plot, the PERMANOVA revealed a borderline significant effect of treatment on overall gene expression when accounting for effects of sampling time and sequencing run.

**TABLE 3:** PERMANOVA results of stomach RNA-sequencing data from early-life control or *H. pylori*-infected mice^*a*^

	*F*-value	*P*-value	*R* ^2^
Treatment	2.7111	0.056	0.02832
Time	23.1117	0.001	0.24140
Run	50.9181	0.001	0.53183

^*a*^ No interactions between factors were found to be significant and were thus removed from the model. Run explains 53.2% of the variation in the data, and time an additional 24.1%. There was a marginally significant effect of treatment, explaining 2.8% of the variation.

To identify differentially expressed genes between treatment groups, DESeq2 analysis was performed with two designs, to analyze the effect across both time points and at the individual time points, controlling for sequencing run. In total, only 13 genes were found to be significantly differentially expressed (*P*_adj_ < 0.05); 4 genes by treatment across both time points (*Gpt, Magix, Mns1,* and *Shisa3*); 3 genes when performing the analysis on only week 1 data (*Ddr, Itpr1,* and *Tnni3*) and 6 genes when performing the analysis on week 2 data (*Dusp1, Exo5, Reg3g, Siah2, Csn2,* and *AI197445*). The overall pattern of expression between groups was the same in the two runs; however, values were generally lower in the Novogene data set (Fig. S3). This was accounted for by controlling for sequencing run in the DESeq2 analyses.

### STRING network revealing enriched pathways

**Fig 8:**
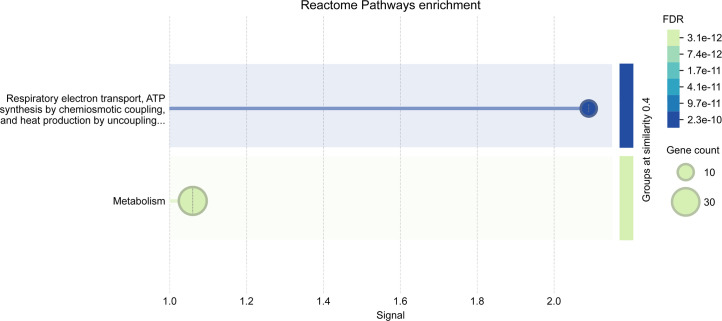
Enriched pathways of upregulated genes (*P*_adj_ < 0.1) in week 1 samples from *H. pylori*-infected mice. Illustration from STRING for signal >1 and merging terms with a similarity ≥0.5.

**Fig 9:**
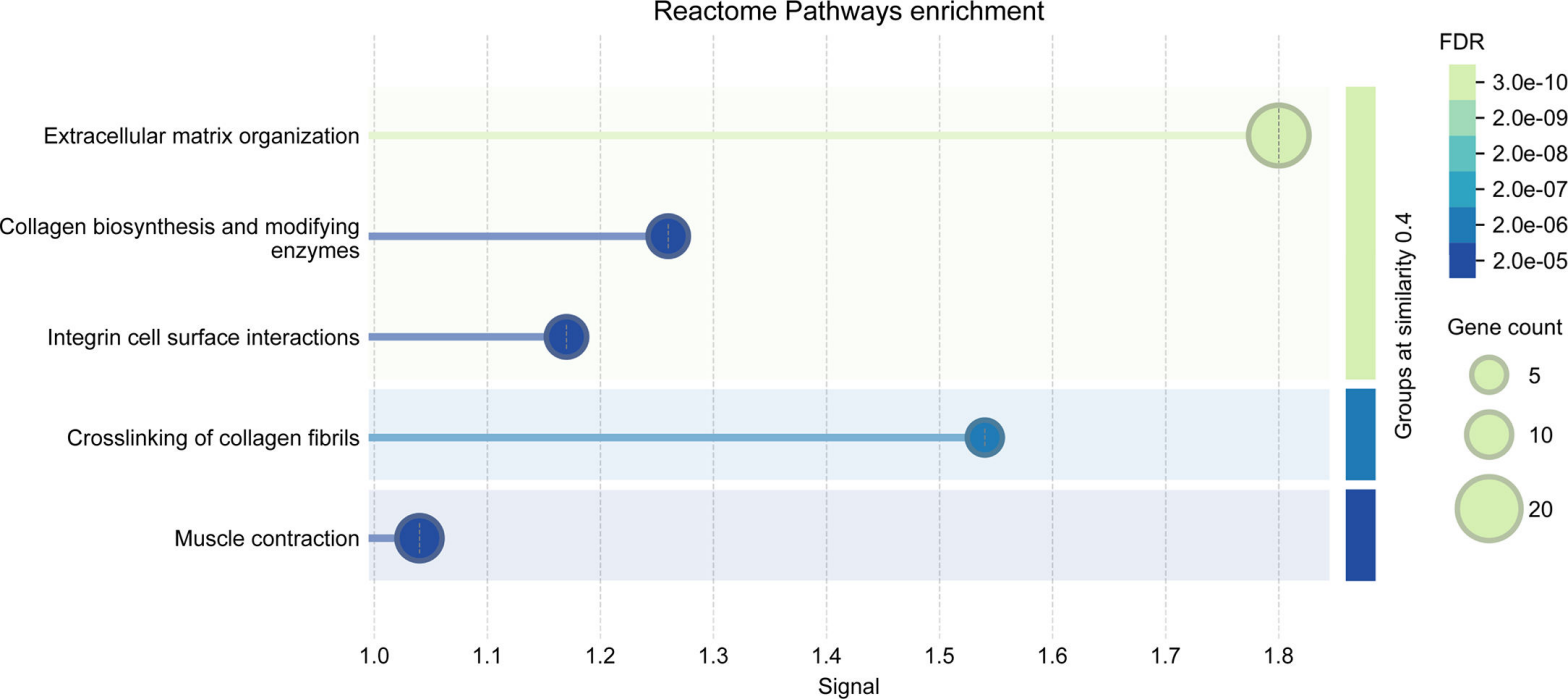
Enriched pathways of downregulated genes (*P*_adj_ < 0.1) in week 1 samples from *H. pylori*-infected mice. Illustration from STRING for signal >1 and merging terms with a similarity ≥0.5.

**Fig 10:**
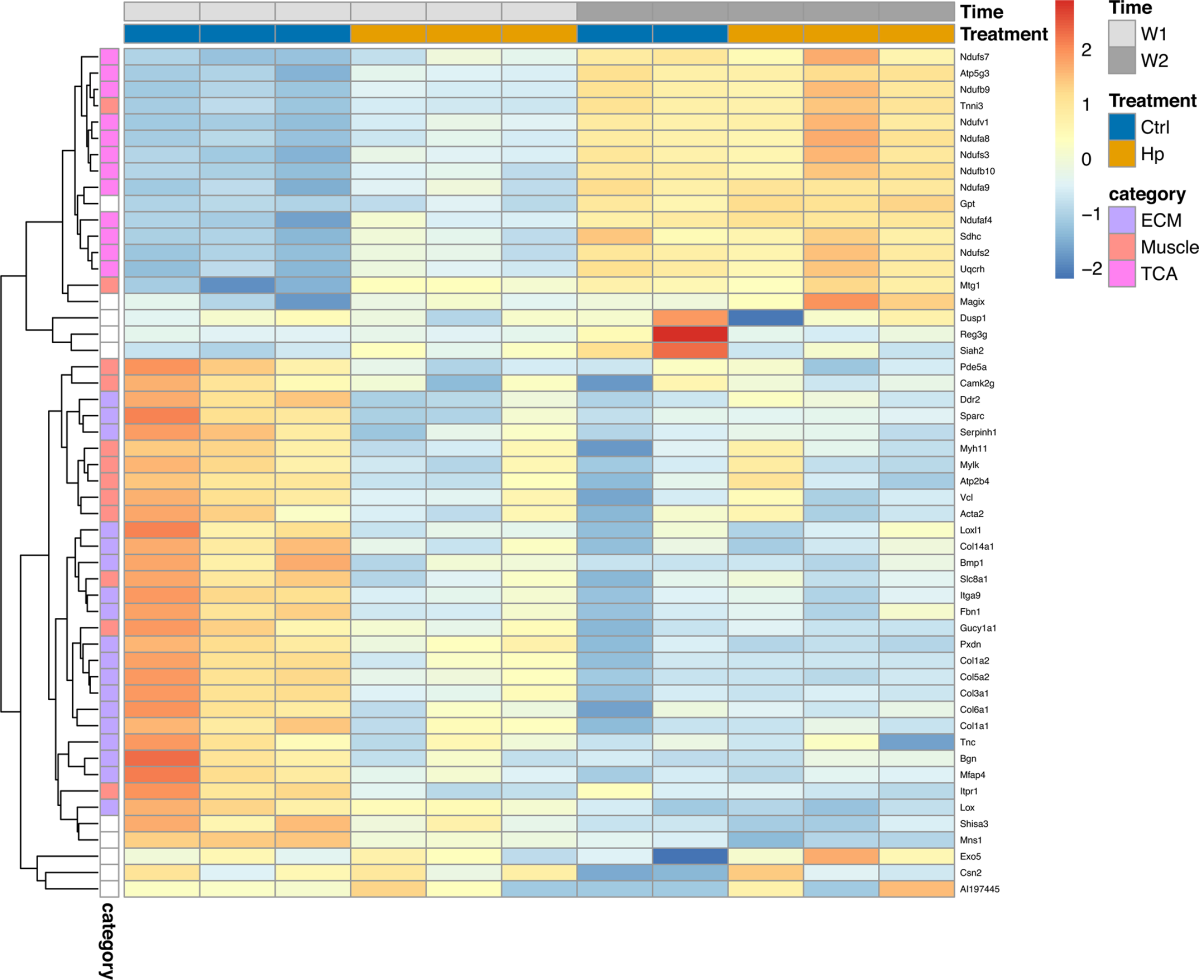
Heatmap of scaled expression of genes that were differentially expressed with a *P*_adj_ < 0.05 or 0.1 and classified as belonging to an enriched Reactome pathway in the STRING analyses (ECM, extracellular matrix; Muscle, muscle contraction; TCA, the citric acid cycle). Only data from Novogene are shown due to the differences in expression levels between sequencing runs. See Fig. S4 for transcriptomic data from Rigshospitalet showing the same patterns.

To identify enriched pathways, we increased the cutoff to a more permissive *P*_adj_ < 0.1 and extracted the DE genes for week 1 (68 up, 116 down), week 2 (5 up, 12 down), and across time points (13 up and 11 down). We found enriched Reactome pathways at a signal cutoff of 1 for genes that were differentially expressed in week 1 after gavage (Fig. 8 and 9). For these genes, the expression in *H. pylori*-infected mice in week 1 resembled that of all mice sampled in week 2 more than that of the control mice in week 1 (Fig. 10). In the upregulated set, 12 genes were classified in the “Citric acid cycle and respiratory electron transport” pathway, and subsets of these genes were further classified as various pathways related to Complex 1 biogenesis and respiratory electron transport (Fig. 8). These and an additional 19 genes were also classified in a broad “Metabolism” pathway. For the majority of these genes, expression was lowest in week 1, with tissue from *H. pylori*-infected mice exhibiting higher expression than that from control mice, and with expression higher and similar between groups in week 2 (Fig. 10).

In the set of genes downregulated by *H. pylori* infection, 18 genes were classified in the “extracellular matrix organization” pathway (including the *Ddr* gene with *P*_adj_ < 0.05), and subsets of these genes were further classified as various pathways related to collagen (Fig. 9). An additional 10 genes (including *Itpr1* with *P*_adj_ > 0.05) were classified in the muscle contraction pathway. While genes related to muscle contraction were generally downregulated, two upregulated genes also belonged to this pathway (including *Tnni3* with *P*_adj_ > 0.05). For the majority of the genes, expression in tissue from control mice was higher in week 1 compared to week 2, and infection with *H. pylori* lowered expression in week 1 to resemble the expression of all mice in week 2 (Fig. 10).

## DISCUSSION

### *H. pylori* is found in the entire stomach in the 1st month of life

We characterized the impact of experimental *H. pylori* infection on the mouse gastrointestinal environment at the dynamic 1st month of life. Stomach morphology and gene expression change radically at this time, as the mice gradually start eating solids around 2 weeks of age (44). We found that the initially low *H. pylori* load increased, with a trend toward a preference for the lower glandular part of the stomach, as previously found (5, 45, 46) (Fig. 1). The upper part that is the forestomach does not contain the glands that house *H. pylori* (45, 46), which fully develop around 3 weeks after gestation (47). In the small tissue sections, we did not distinguish between the antrum and corpus part of the lower glandular stomach, where *H. pylori* early in infection has been found to reach higher loads in the antrum (46). The qPCR results do not allow for distinguishing between live and dead bacteria. Confirming the viability of the present *H. pylori* bacteria would require growing the bacteria for counting of colony-forming units. However, the ongoing mucosal clearance is expected to remove bacteria that do not colonize crypts (45). The drop in bacterial load we observed from 2 days to 1 week after gavage (Fig. 1) also suggests a wash-out of bacteria that do not closely associate with the host tissue.

The relative abundance of *H. pylori* in the stomach 1 and 2 weeks after gavage was low and never exceeded 3%. This is in contrast to mean levels at 26% in adult mice following the same gavage treatment early in life (28). We did not detect the bacteria in the small intestine by qPCR or in the colon by 16S rDNA sequencing (Fig. 4). The gastric pH in young mice is higher than that of adult mice (around pH 4–5 at 1 week compared to <3 [48] or around 3 [49] in adults). The higher gastric pH reduces the competitive edge in the stomach that acid tolerance provides *H. pylori* with. While the neonatal immune system, on one hand, is tolerant of microbial associates (12), maternal antibodies and milk oligosaccharides may bind and limit bacterial growth (15).  

### *H. pylori* infection shapes the stomach and colon microbiomes despite a low relative abundance

The stomach and colon microbiomes were strikingly similar between samples from the same week, and alpha diversity was low. One week after gavage, the mice were 2 weeks old and primarily nursing, which is reflected in the microbiome dominated by lactic acid bacteria. There has been limited research done on neonatal stomach microbiomes; one study found *Lactobacillus* dominance but did not compare with the gut microbiome (50). *Ligilactobacillus* has previously been found to dominate the intestinal microbiome in neonatal mice, driven by the uniform milk diet, and *Staphylococcaceae* genera have likewise been sampled from neonatal mice likely originating from maternal skin, oral, or vaginal sites (17). The similarity between stomach and colon microbiomes in week 1 may be related to the higher gastric pH in neonatal mice, making the conditions along the gastrointestinal tract more uniform. The alpha diversity rose drastically between week 1 and week 2 (Fig. 2). The gastrointestinal microbiome is developing fast in this early stage of life, and 2 weeks after gavage, the mice were ingesting more solid food. At this time, mice also become coprophagic, which means that they ingest their own and nestmates’ stool. Thus, the detected bacteria are not necessarily colonizing the stomach but may just be ingested, and this could in part explain the observed rise in diversity of the stomach microbiome and its similarity to that of the colon.

Two weeks after infection, alpha diversity was significantly reduced in colon samples from *H. pylori*-infected mice, and a similar trend was observed for stomach samples (Fig. 5), despite *H. pylori* only reaching a median prevalence of 0.64% in week 2 (Fig. 4B). The lower alpha diversity of *H. pylori*-infected mice was thus not driven by a high prevalence of *H. pylori* in these samples, as has been found in humans (7, 9). This points to *H. pylori* being able to alter the diversity in both the colon and the stomach indirectly. In a study where mice were infected with *H. pylori* at 4–6 weeks of age, beta diversity in the ileum and cecum was significantly altered between the infected and control groups (8). We did not profile the immune response to infection in this study, but the indirect effects are hypothesized to occur through modulation of the immune response, affecting the microbiome composition (8). Pups from the *H. pylori*-infected litter 15 had a large proportion of bacteria belonging to *Lachnoclostridium* and *Bacteroides,* causing low alpha diversity. Pups from the same litter had a higher prevalence of *Enterobacter* in week 1 colon samples that may represent a disturbance of the microbiota. The two litters were infected with *H. pylori* on the same days with the same inoculum, so this should not have contributed to the differences we observe.

*Akkermansia muciniphila* bacteria reached significantly higher relative abundances in both the stomach and colons of *H. pylori*-infected mice (Fig. 4A). In our recent study of early-life *H. pylori* infection in mice, we also found a significant bloom of *Akkermansia muciniphila* in *H. pylori*-infected mice on a high-fat diet, when present in the maternal or littermate microbiome (28). Here, we do not know the *A. muciniphila* status of the dams. *A. muciniphila* is mucus-degrading and has gained attention as a probiotic, as studies find that it can contribute to weight loss (51–53) and improve the efficacy of immunotherapy in some cancer treatments (54). However, it may also act as an opportunist when the gut microbiota has been perturbed, e.g., by antibiotics (55, 56), and we found its abundance to be highest in the *H. pylori*-infected mice that had a worse metabolic profile compared to controls (28).

### Gene expression of key stomach development processes affected early in infection

Genes in pathways related to extracellular matrix formation, smooth muscle contraction, and metabolism were differentially expressed in *H. pylori*-infected mice 1 week after gavage. These processes have previously been found to be affected by *H. pylori* infection in cell and animal models and in human biopsies (57, 58). The extracellular matrix provides structure to the stomach tissue and serves as a medium for intercellular communication and a scaffold for the stomach muscles that facilitate gastric motility. Tissue invasion and disruption by *H. pylori* can expose the underlying extracellular matrix, and the bacteria adhere to components of this structure such as collagen (59). In rats, *H. pylori* infection causes increased collagen deposition that invades the submucosa muscle fibers and a decrease in muscle contraction force and rate (60). The associated stomach immobility and delayed stomach emptying have been implicated in patients experiencing dyspepsia (61), while gastric emptying was accelerated in one study of *H. pylori* infection in children (62).

Expression of these key processes in infected mice shifted away from that of control mice toward that of all mice sampled the subsequent week (Fig. 10). The stronger signal of infection on expression in week 1 may be caused by the high bacterial load in the 1st week from the gavage inoculum (Fig. 1) or represent the acute response to infection. The two time points we sampled have been characterized as distinct phases of stomach development, marked specifically by a switch from high expression of focal adhesion structures and epithelial receptors targeting the extracellular matrix toward increased metabolism and maturation (44). Whether the altered expression we observed in infected mice compared to controls, resembling that of 1 week older mice, reflects faster tissue development or impairment of growth is unclear. Additional time points and histological data are needed to explore this. The carcinogenic effects of *H. pylori* involve cell proliferation by inducing stemness properties (63). Tumorigenesis of diffuse gastric cancer was found to share cell proliferation markers of early stomach development (44), and the expression differences we observe may reflect brief early reprogramming, with lifelong effects.

### Strengths and limitations of experimental design

By sampling at multiple time points, we were able to capture dynamic changes in the microbiome and gene expression over time, enhancing our understanding of the impact of early-life *H. pylori* infection on host development. The stomach microbiota of mice has been studied less than fecal microbiomes and especially that of neonates (50). Transcriptomics was performed in two batches with different approaches, which introduced extensive technical variation. This likely made it more difficult to detect more subtle differences in expression between groups. Additional time points for the microbiome and gene expression analyses, in combination with histology, would provide more insight into how infection shapes development. We further observed litter effects in the microbiome analyses within the *H. pylori* group, and ideally, samples from more litters in both groups had been analyzed. As controls for the *H. pylori* treatment, we used mice gavaged with the rich bacterial growth media, which likely in itself affects the microbes and host in the short term. It would be interesting to also include untreated controls to differentiate this effect.

## Data Availability

Microbiome and RNA sequencing data is available as NCBI BioProject PRJNA1219814.

## SUPPLEMENTAL MATERIAL

Fig. S1 iai.00250-25-s0001.pdf Dissection workflow.

Fig. S2 iai.00250-25-s0002.eps Rarefaction curve.

Fig. S3 iai.00250-25-s0003.eps Gene expression.

Fig. S4 iai.00250-25-s0004.eps Heatmap.

Supplemental material iai.00250-25-s0005.docx Supplemental legends; Table S1.

ASM does not own the copyrights to Supplemental Material that may be linked to, or accessed through, an article. The authors have granted ASM a non-exclusive, world-wide license to publish the Supplemental Material files. Please contact the corresponding author directly for reuse.
