# Supplementary figures and images for: Neonatal infection with Helicobacter pylori affects stomach and colon microbiome composition and gene expression in mice
Source: Infect Immun. 2025 Sep 22;93(10):e00250-25. doi: 10.1128/iai.00250-25 (PMC12519789; doi:10.1128/iai.00250-25)

A

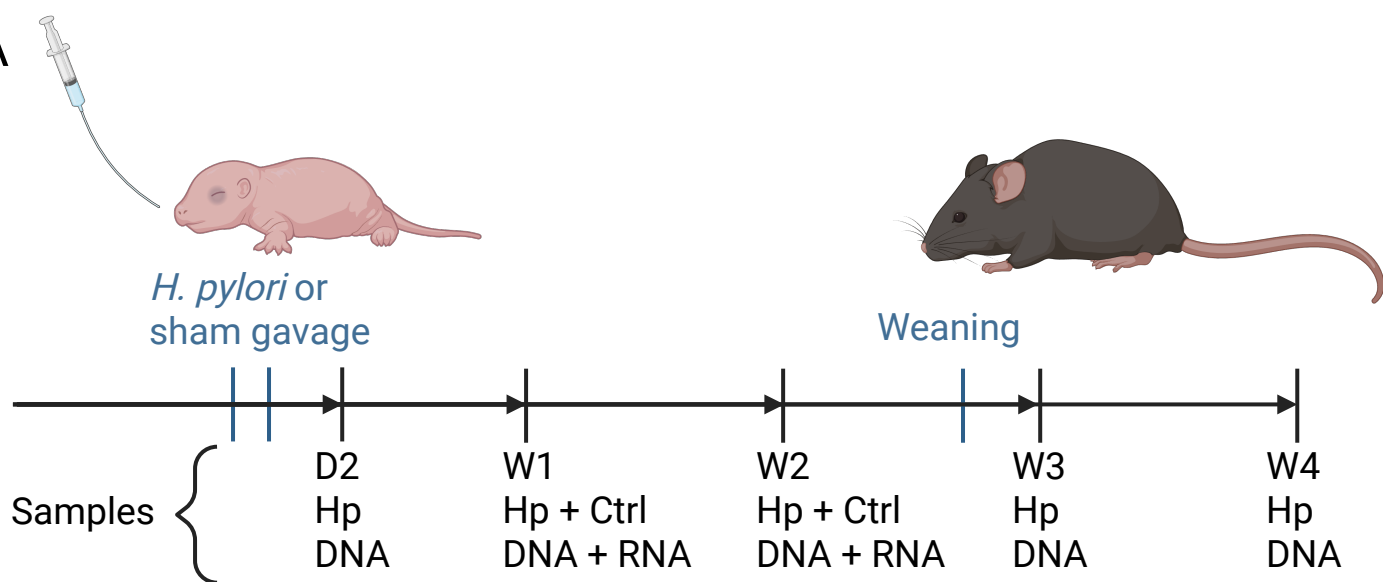

B

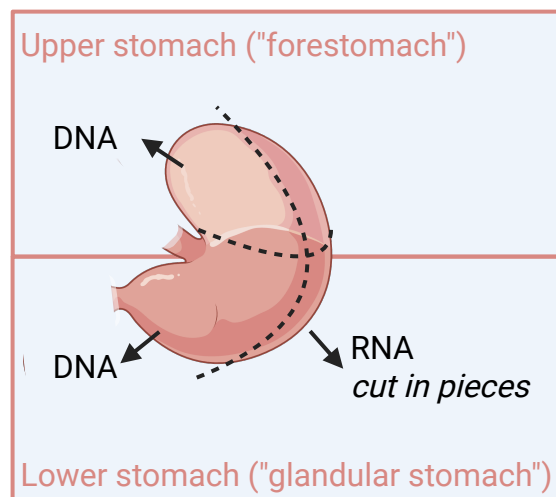

Supplement: Fig. S1 — Dissection workflow. [file iai.00250-25-s0001.pdf]
