# Supplementary material for: Neonatal infection with Helicobacter pylori affects stomach and colon microbiome composition and gene expression in mice
Source: Infect Immun. 2025 Sep 22;93(10):e00250-25. doi: 10.1128/iai.00250-25 (PMC12519789; doi:10.1128/iai.00250-25)
Supplement: Supplemental material — Supplemental legends; Table S1. [file iai.00250-25-s0005.docx]

**Supplementary materials**

**Supplementary material legends**

**Fig. S1:** Animal experiment outline (A) and workflow for dissection of stomach tissues for RNA and DNA extraction (B) Created in BioRender. Andersen, S. (2025) https://BioRender.com/cx0p4r8

**Fig. S2:** Rarefaction curves depicting sequencing depth as sample size on the x-axis and ASV count on the y-axis for the stomach (S) and colon (C) samples.

**Fig. S3:** Stomach tissue gene expression of genes related to the extracellular matrix from samples collected one week after gavage (W1) and two weeks after gavage (W2) from mice infected with *H. pylori* (yellow) or treated with a control solution (blue). Expression levels, given as gene counts normalised by gene length, varied between sequencing runs (open circles = Novogene, filled circles = Rigshospitalet). However, for the majority of genes the pattern between treatment was the same, with overall higher expression levels in the Rigshospitalet data set. Note the different scales on the y-axis between plots.

**Fig. S4:** Heatmap of scaled expression of genes that were differentially expressed with a p_adj_ < 0.05, or 0.1 and classified as belonging to an enriched Reactome pathway in the STRING analyses (ECM = extracellular matrix, Muscle = muscle contraction, and TCA = the citric acid cycle). Only data from Rigshospitalet is shown, due to the differences in expression levels between sequencing runs (see **Fig. 10** for the Novogene dataset).

**Table S1**

Overview of mice included in the study and indication of sampling time relative to last infection with *H. pylori* (Hp) or control (Ctrl), and relative to weaning as well as indications of what analyses have been applied**.** For mice sacrificed before weaning, sex was estimated after the RNAseq analysis based on Xist gene expression, which is only expressed in females.

| **Sampling time** | |  |  |  |  |  |
| --- | --- | --- | --- | --- | --- | --- |
| **Time since**  **last infection** | **Time since**  **weaning** | **Treatment** | **Litter** | **Sex** | **Stomach and SI Hp quantification by qPCR** | **Stomach and colon 16S seq and stomach RNAseq** |
| 2 days | NA | **Hp** | 4a | NA | Yes | - |
| 2 days | NA | **Hp** | 4a | NA | Yes | - |
| 2 days | NA | **Hp** | 4a | NA | Yes | - |
| 2 days | NA | **Hp** | 4a | NA | Yes | - |
| 2 days | NA | **Hp** | 4a | NA | Yes | - |
| 2 days | NA | **Hp** | 4a | NA | Yes | - |
| 1 week | NA | **Hp** | 6a | F | Yes | Yes |
| 1 week | NA | **Hp** | 6a | M | Yes | Yes |
| 1 week | NA | **Hp** | 6b | F | Yes | Yes |
| 1 week | NA | **Hp** | 15a | M | Yes | Yes |
| 1 week | NA | **Hp** | 15a | M | Yes | Yes |
| 1 week | NA | **Hp** | 15b | M | Yes | Yes |
| 1 week | NA | Ctrl | 14a | F | Yes / neg control | Yes |
| 1 week | NA | Ctrl | 14a | F | - | Yes |
| 1 week | NA | Ctrl | 14a | F | - | Yes |
| 1 week | NA | Ctrl | 14b | F | - | Yes |
| 1 week | NA | Ctrl | 14b | F | - | Yes |
| 1 week | NA | Ctrl | 14b | M | - | Yes |
| 2 weeks | NA | **Hp** | 6a | M | Yes | Yes |
| 2 weeks | NA | **Hp** | 6a | M | Yes | Yes |
| 2 weeks | NA | **Hp** | 6b | F | Yes | Yes |
| 2 weeks | NA | **Hp** | 15a | M | Yes | Yes |
| 2 weeks | NA | **Hp** | 15a | F | Yes | Yes |
| 2 weeks | NA | **Hp** | 15b | M | Yes | Yes |
| 2 weeks | NA | Ctrl | 14a | M | Yes / neg control | Yes |
| 2 weeks | NA | Ctrl | 14a | M | - | Yes |
| 2 weeks | NA | Ctrl | 14a | F | - | Yes |
| 2 weeks | NA | Ctrl | 14b | NA | - | Yes, but RNAseq failed |
| 2 weeks | NA | Ctrl | 14b | M | - | Yes |
| 2 weeks | NA | Ctrl | 14b | F | - | Yes |
| 3 weeks | 2 days | **Hp** | 15a | F | Yes | - |
| 3 weeks | 2 days | **Hp** | 6b | F | Yes | - |
| 3 weeks | 2 days | **Hp** | 6b | F | Yes | - |
| 3 weeks | 2 days | **Hp** | 15a | M | Yes | - |
| 3 weeks | 2 days | **Hp** | 6a | M | Yes | - |
| 3 weeks | 2 days | **Hp** | 6a | M | Yes | - |
| 4 weeks | 10 days | **Hp** | 15b | F | Yes | - |
| 4 weeks | 10 days | **Hp** | 6a | F | Yes | - |
| 4 weeks | 10 days | **Hp** | 15a | M | Yes | - |
| 4 weeks | 10 days | **Hp** | 15b | M | Yes | - |
| 4 weeks | 10 days | **Hp** | 6b | M | Yes | - |
| 4 weeks | 10 days | **Hp** | 6b | M | Yes | - |
